# Standardized Testing and Quantitative Safety Assessment for Upper Limb Rehabilitation Robots: A Bionic Robotic Platform and Integrated Evaluation Framework

**DOI:** 10.3390/biomimetics11070456

**Published:** 2026-07-01

**Authors:** Yuheng Jiang, Yanchen Du, Shengli Luo, Xiaolong Shu, Qingzhuo Yuan, Hongliu Yu

**Affiliations:** 1 School of Health Science and Engineering, University of Shanghai for Science and Technology, Shanghai 200093, China; 243352375@st.usst.edu.cn (Y.J.);; 2 Shanghai Engineering Research Center of Assistive Devices, Shanghai 200093, China

**Keywords:** upper limb rehabilitation robot, testing platform, bionic upper limb, control system framework, safety assessment

## Abstract

To address the lack of standardized safety assessment tools for upper-limb rehabilitation robots, this study developed an integrated testing platform and a quantitative safety assessment framework, demonstrated with FlexoArm1 as a proof-of-concept. A 6-degree-of-freedom bionic arm equipped with multiple sensors was constructed, and a fuzzy PID control algorithm was employed to improve motion trajectory tracking accuracy. A fuzzy multi-criteria safety assessment model was established by combining the Analytic Hierarchy Process (AHP) with the entropy weight method. Experiments were conducted on the rehabilitation robot FlexoArm1. The platform reliably replaced human subjects in range-of-motion testing, interactive torque measurement (peak torque approximately 6.2 N·m in fully active mode), and spasticity simulation, with angular data showing close agreement with Inertial Measurement Unit (IMU) measurements. The assessment model assigned a comprehensive safety score of 70.23 to the tested device, successfully identifying weaknesses in fault detection capability and structural safety design. The proposed bionic-arm-based testing platform and the accompanying safety assessment methodology provide practical tools and a quantitative basis for standardizing safety evaluation and guiding design optimization of upper-limb rehabilitation robots.

## 1. Introduction

Stroke is the third leading cause of disability globally, and its rising disease burden has made it a major public health concern, contributing substantially to both mortality and long-term disability [1,2]. As the population ages, the number of stroke survivors continues to rise. These patients not only experience neurobiological dysfunction but also face a substantially elevated risk of post-stroke depression [3,4]; approximately one-third of stroke survivors are affected by depression, which considerably impedes rehabilitation outcomes and quality of life [3]. This dual burden of physical and psychological impairment imposes heavy human and economic costs on patients, families, and the broader healthcare system. Encouragingly, repetitive, task-oriented physical rehabilitation can partially restore motor function in stroke survivors, offering a realistic pathway toward functional recovery [5]. However, traditional rehabilitation therapy relies primarily on manual operation by physiotherapists. This approach is labor-intensive, difficult to scale, and struggles to meet the growing clinical demand.

Upper-limb rehabilitation robot technology has therefore emerged as a promising solution: by combining robotics with rehabilitation medicine, these devices provide patients with efficient, controllable, and repeatable training, and they have become an important focus of modern rehabilitation engineering [6]. Despite this progress, upper-limb rehabilitation robots still face serious safety challenges in clinical practice. Rigid-joint robots, for example, struggle to absorb the high-frequency force fluctuations that arise from natural human movement; when a patient attempts to move actively, high mechanical stiffness and low back-drivability can cause stumbling or discomfort [7]. Current safety testing relies predominantly on manual clinical trials, which suffer from high subjectivity, poor data quantification, limited repeatability, and the absence of a systematic, standardized assessment framework [8].

Clinical studies and systematic reviews on upper-limb rehabilitation robots have identified skin and soft-tissue injuries as commonly reported adverse events at the human–robot interface [9,10,11], changes in physiological parameters [12], and subjective pain ratings [13]. Mechanical indicators, including joint range-of-motion limits and driving torque control, have also been recognized as key safety metrics [14]. However, these studies are mostly based on human trials, which limits the potential for objective, quantitative assessment. With regard to testing equipment, Akiyama et al. [15] developed the Instrumented Cuff, a device capable of measuring mechanical parameters at the human–robot interface. Toth et al. [16] proposed an anthropometrically adjustable, sensorized dummy limb for testing ROM safety functions. Shah et al. [17] designed a bio-mimicking multimodal tactile sensor for upper-limb prostheses that combines differential capacitive (static) and piezoelectric (dynamic) sensing, with operating frequencies matching those of targeted human mechanoreceptors. Despite these contributions, standardized testing platforms specifically designed for comprehensive safety evaluation of upper-limb rehabilitation robots remain scarce. Existing equipment exhibits significant deficiencies in reproducing the multi-joint kinematic characteristics of the human upper limb, performing synchronous multi-parameter acquisition, and integrating quantitative risk assessment models.

Furthermore, several international standards provide frameworks for the safety evaluation of medical and rehabilitation robots but remain underutilized in the context of upper-limb rehabilitation devices. IEC 80601-2-78 [18] specifies particular requirements for the basic safety and essential performance of medical robots used for rehabilitation, emphasizing risk management processes in line with ISO 14971 [19]. ISO 13482 [20] establishes safety requirements for personal care robots, including physical human–robot interaction considerations applicable to wearable rehabilitation devices. ISO/TS 15066 [21] supplements ISO 10218 [22] by providing guidance on collaborative robot operation, including force and pressure limits for human contact, which are directly relevant to the interaction force thresholds between rehabilitation robots and patients’ limbs. Despite these established standards, their application to upper-limb rehabilitation robots remains limited due to the lack of standardized testing platforms and quantitative metrics tailored to the specific kinematic and kinetic characteristics of the human upper limb. Addressing this gap—the disconnect between existing regulatory frameworks and the practical tools needed to generate compliant, quantitative evidence—is a central motivation for the present work.

To address the aforementioned research gaps, this study aims to design a testing platform for upper limb rehabilitation robots based on a bionic upper limb and establish a corresponding safety assessment model. By simulating the kinematic characteristics and mechanical responses of the human upper limb, this platform can replace human trials, enabling quantitative testing of key indicators such as device workspace, interaction torque, and spasticity protection function. The research content covers the mechanical structure design of the testing platform, control system development, control algorithm optimization, and the construction of a fuzzy multi-criteria safety assessment model based on the AHP-Entropy Weight Method. The innovations are mainly reflected in two aspects:

(1) Proposing a testing platform centered on a bionic upper limb, capable of accurately reproducing human upper limb motion trajectories and integrating multiple sensors for real-time data acquisition;
(2) Introducing an assessment method that integrates subjective and objective weights, significantly enhancing the scientific rigor and reliability of safety evaluation.

This work provides both a theoretical basis and practical tools for the safety design of upper-limb rehabilitation robots, and is expected to support the development and refinement of related technical standards. The experimental results confirm that the platform performs well in workspace simulation, human motion reproduction, and spasticity state testing, thereby laying a foundation for subsequent device optimization and clinical translation.

## 2. Materials and Methods

The bionic upper-limb testing platform was designed using Siemens NX for 3D mechanical modeling. The control system firmware was developed in C using Keil MDK-ARM V5.36 and deployed on the STM32F407ZGT6 microcontroller running FreeRTOS V10.4. The human–machine interaction software was developed with Qt Creator 8.0.0 (C++) on Ubuntu 22.04 LTS. Control algorithm simulations were performed in MATLAB R2023a. Statistical analyses, including Bland–Altman analysis, were conducted using MATLAB R2023a. Entropy weight calculations and fuzzy comprehensive evaluation were implemented via custom MATLAB scripts. The testing platform prototype was fabricated using a combination of 3D-printed components, CNC-machined aluminum alloy parts, and off-the-shelf electromechanical components.

### 2.1. Overall Mechanical Design

**Figure 1:**
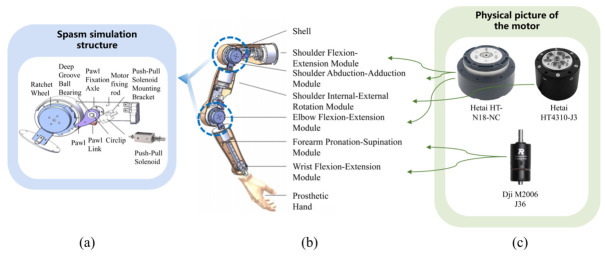
(**a**) Schematic diagram of the spasticity simulation structure. (**b**) Main mechanical structure of the testing platform. (**c**) Physical picture of the motors.

The main mechanical structure of the testing platform is shown in Figure 1b. The platform features six degrees of freedom (DOFs) that replicate the principal joint motions of the human upper limb: shoulder flexion/extension, adduction/abduction, and internal/external rotation; elbow flexion/extension; forearm pronation/supination; and wrist flexion/extension. A prosthetic hand and anatomical shell complete the assembly.

Upper limb flexion spasticity is a common spasticity pattern in hemiplegic patients, characterized by increased muscle tone in the flexor muscle groups and relatively weakened or normal muscle tone in the extensor muscle groups of the upper limb. This causes the patient’s upper limb to maintain a persistent flexed posture, such as shoulder flexion, internal rotation and adduction, elbow flexion, wrist flexion, and finger flexion and adduction. This spasticity pattern not only affects the patient’s daily life but may also lead to complications such as pain, muscle fatigue, and joint deformities [23,24]. Therefore, a spasticity simulation structure was designed to simulate the adducted shoulder and flexed elbow spasticity state in hemiplegic patients. Figure 1a shows a schematic diagram of the spasticity simulation structure at the shoulder adduction/abduction and elbow flexion/extension degrees of freedom. It also includes components such as push-pull solenoids, solenoid fixtures, pawls, pawl fixing shafts, deep groove ball bearings, circlips, and pawl connecting rods.

To ensure that the bionic arm approximates the passive mechanical response of a human upper limb, the segment masses, lengths, center-of-mass (COM) positions, and moments of inertia were designed to match the anthropometric properties of a 50th-percentile adult male, as reported by Winter [25] and De Leva [26]. The design-target parameters for each segment are summarized in Table 1. Because passive joint stiffness and damping are inherently nonlinear and angle-dependent [27,28], the stiffness and damping coefficients in Table 1 represent linearized nominal estimates at the mid-range of joint motion; these values serve as initial design references for the bionic arm control system rather than exact physiological replicas.

### 2.2. Overall Control System Scheme

**Figure 2:**
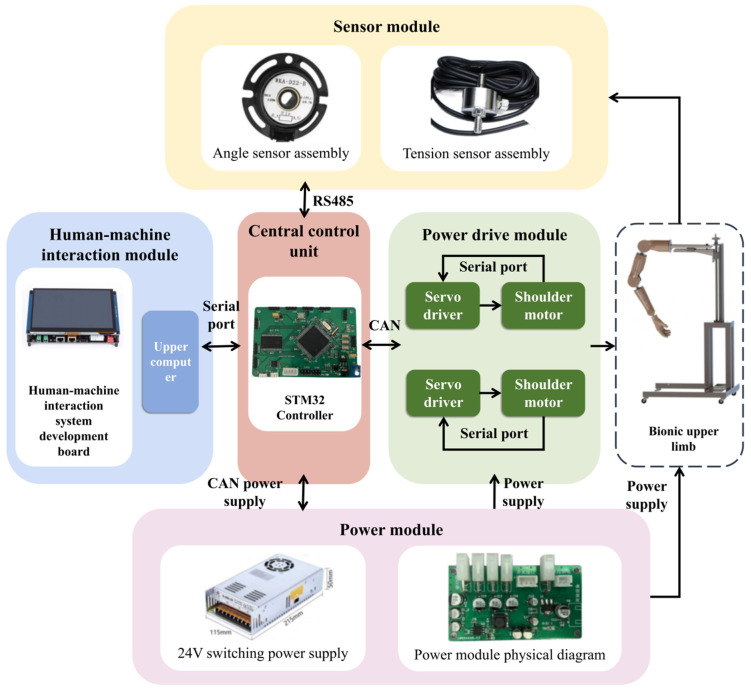
Overall control system framework.

The control system for the upper limb rehabilitation robot test platform employs a modular, hierarchical architecture centered on a central controller. This design integrates power management, sensor data acquisition, human–machine interaction, and drive modules to ensure reliable, real-time, and safe operation. Data exchange follows standardized protocols (e.g., CANopen, Modbus-RTU). High-precision sensors collect motion parameters like joint angles and torque, while the software integrates kinematic modeling with a fuzzy PID control algorithm to achieve precise bionic motion simulation. This scheme enables the quantitative assessment of safety indicators –such as workspace and interaction forces –to support standardization, replacing manual testing methods. The overall system framework is shown in Figure 2.

The power module accepts 220 V AC input, which is converted via switching power supplies to multiple protected DC outputs (24 V, 5 V, 3.3 V) to ensure stable operation. The sensor acquisition module employs potentiometric angular sensors (e.g., WKA-D22-B) and high-precision tension sensors (e.g., DSMH-103). Their analog signals are digitized and transmitted in real-time to the central controller via the RS485 bus using the Modbus-RTU protocol. The human–machine interface, developed on an embedded Linux system with the LVGL graphics library, utilizes a 7-inch touchscreen for parameter configuration, data visualization, and report generation. The central controller, based on an STM32F407ZGT6 microprocessor running the FreeRTOS real-time operating system, handles multi-tasking (e.g., data parsing, motor control) and communicates with the motor set via a CAN bus to achieve closed-loop motion control of all six joints. Each joint is actuated by an independent DC motor with a planetary gear reducer and is equipped with built-in angle and torque sensors for real-time data acquisition. The wrist flexion/extension joint employs a transmission mechanism and provides a range of motion from −65° (inward) to +65° (outward), with the motor mounted on a fixing plate attached to the forearm linkage. The forearm pronation/supination joint is similarly motor-driven, enabling the platform to replicate the full six-degree-of-freedom kinematic chain of the human upper limb.

**Figure 3:**
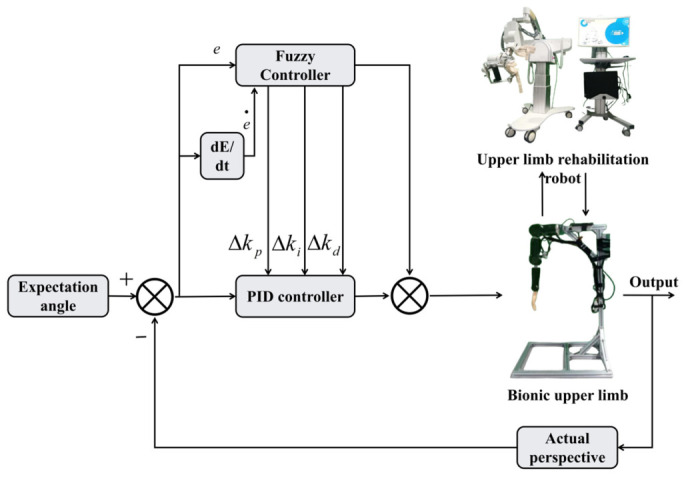
Fuzzy PID control structure diagram.

The control scheme is based on the bionic upper limb kinematics. A simplified model is established using the D-H parameter method, with derived forward and inverse kinematic equations providing the theoretical foundation for trajectory planning. A fuzzy PID controller is implemented to enhance system response speed and disturbance rejection by dynamically tuning its parameters. MATLAB/Simulink simulations demonstrate its superiority over traditional PID control in terms of reduced overshoot and improved stability. This controller dynamically adjusts the PID parameters based on the instantaneous error and its rate of change. The adjustment formula is as follows:

$$K_{p}\;={\;K}_{p0}\;+\;\Delta K_{p}$$

(1) $$\begin{array}{c} K_{i}={\;K}_{i0}+\Delta K_{i} \end{array}$$

$$K_{d}=c+\Delta K_{d}$$

where $K_{p0},K_{i_{0}},K_{d_{0}}$ are the initial PID parameters, and $\Delta K_{P}$, $\Delta K_{i}$, $\Delta K_{d}$ are the PID parameter variations output by the fuzzy controller. Based on the upper limb rehabilitation robot testing platform, this study uses fuzzy PID control to complete active training mode tests for different devices under test. The error between the target joint position and the actual angular position, as well as the derivative of the error, are used as inputs. The control structure diagram is shown in Figure 3.

According to the principles of fuzzy control, the control parameters of the testing platform are converted into fuzzy sets. To simplify complexity, the fuzzy sets for e, $\dot{e}$, $\Delta K_{P}$, $\Delta K_{i}$, $\Delta K_{d}$ are defined as: (“NB”, “NM”, “NS”, “ZO”, “PS”, “PM”, “PB”) corresponding to the fuzzy universe range of (−3, −2, −1, 0, 1, 2, 3). Values outside the range [−3, 3] are normalized into the interval [−3, 3].

Based on the motion characteristics of the testing platform’s bionic upper limb, during the lifting phase, the system needs to overcome gravity and the interaction force from the wearable upper limb rehabilitation robot. In this case, if an error occurs, a larger $\Delta K_{P}$ and a smaller $\Delta K_{d}$ are needed. Conversely, during the downward swing phase of the bionic arm, the error and error change rate are in the same direction, requiring a smaller $\Delta K_{P}$ and a larger $\Delta K_{d}$ for parameter correction. Based on the above analysis, corresponding fuzzy rules were established in this study, as shown in Table 2.

**Figure 4:**
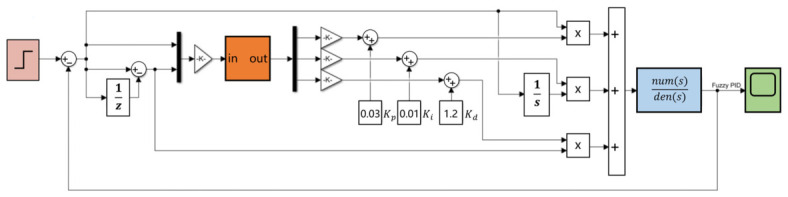
Simulink simulation model of fuzzy PID controller.

**Figure 5:**
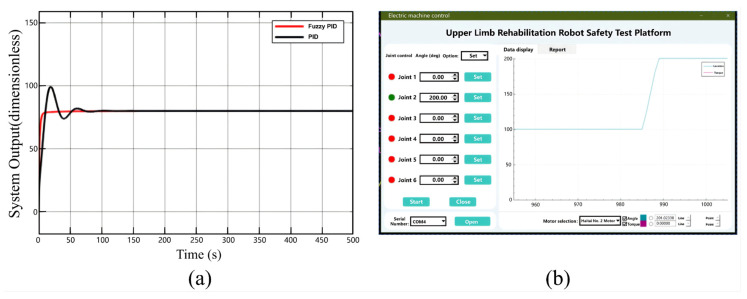
(**a**) Comparison of fuzzy PID and traditional PID control results. (**b**) Human–machine interaction interface of the testing platform.

Triangular membership functions define the mapping between fuzzy sets and the universe, with the center of gravity method employed for defuzzification to yield precise control outputs. The fuzzy PID controller was simulated in MATLAB. Figure 4 shows the Simulink model structure, which includes the fuzzy logic controller block, the PID controller block, and the controlled plant model. The plant transfer function $G(s)=\frac{1}{Js^{2}+Bs}$ was used as a representative second-order model of a DC motor-driven joint with inertial load and viscous friction, approximating the dynamics of a single bionic arm joint. For the simulation scenario, a step reference input was applied to the controlled plant, corresponding to a target elbow joint angular position. In Figure 5a, the *y*-axis represents the system output in arbitrary units (System Output, a.u.; range: 0–150), and the *x*-axis shows elapsed simulation time in seconds. Both the fuzzy PID (red curve) and traditional PID (black curve) controllers converge to the same steady-state value. The simulation window was extended to 500 s solely to illustrate the long-term steady-state convergence properties of both controllers; the transient behavior within the first 50–100 s—during which the fuzzy PID controller exhibits negligible overshoot, rapid settling (within approximately 50 s), and minimal oscillation compared with the pronounced overshoot and prolonged oscillation of the traditional PID controller—is representative of the dynamic response expected during actual rehabilitation motion trajectories. The fuzzy PID controller was compared to a traditional PID controller under identical initial parameter settings.

The simulation results demonstrate that the fuzzy PID controller outperforms the traditional PID controller across key dynamic performance indicators, including faster response speed and lower peak overshoot.

### 2.3. Human–Machine Interaction System Software Design

The human–machine interaction software for the test platform was developed using the Qt framework (Qt Creator 8.0.0, C++) to enable remote control and data visualization through a portable graphical interface. The system comprises three functional modules: (i) data communication, which handles serial-port-based command and data synchronization with the lower-level controller; (ii) interactive control, which manages parameter adjustment and CAN command transmission via Qt’s signal–slot mechanism; and (iii) data visualization, which provides real-time plotting of torque and joint angle data using Qt Charts. The interface is organized into three areas: control (parameter setting), data visualization (real-time charts), and test report (safety evaluation results). Designed with an emphasis on real-time performance and usability, the software employs asynchronous communication and multi-threading to minimize latency, while its modular architecture facilitates maintainability and future extension. The interface layout is shown in Figure 5b.

## 3. Safety Assessment Decision Model

The safety assessment of upper-limb rehabilitation robots requires a structured decision model capable of analyzing, evaluating, and quantifying the likelihood and consequences of hazardous events under specific operating conditions [29]. This section presents a comprehensive assessment model that integrates the Analytic Hierarchy Process (AHP), the Entropy Weight Method, and Fuzzy Comprehensive Evaluation. AHP decomposes a multi-objective decision problem into a hierarchical structure of factors and determines the relative importance of each factor through pairwise comparisons [30,31]. The Entropy Weight Method, grounded in information entropy theory, derives objective weights that reflect the degree of dispersion and the information content of each indicator [32]. In this study, AHP is used to obtain subjective weights from expert opinion, while the Entropy Weight Method provides objective weights from measurement data. The two sets of weights are then fused into optimal combination weights, and the final safety assessment result is derived via fuzzy comprehensive evaluation.

### 3.1. Upper Limb Rehabilitation Robot Safety Evaluation System

**Figure 6:**
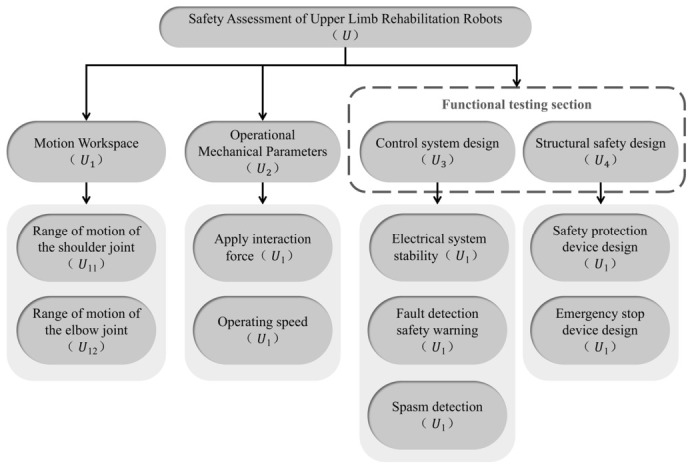
Safety assessment decision system for upper limb rehabilitation robots.

The safety assessment framework for the upper limb rehabilitation robot is structured into four categories: Motion Workspace, Operational Mechanical Parameters, Control System Design, and Structural Safety Design. Specific factors include: shoulder and elbow range of motion (Motion Workspace); interaction force and motion speed (Operational Mechanical Parameters); electrical stability, fault detection, safety warnings, and spasticity detection (Control System Design); and safety protection and emergency stop device design (Structural Safety Design). Based on these indicators, the safety assessment decision system is established as shown in Figure 6.

Within the safety assessment framework, the control system and structural safety design criteria fall under functional testing, encompassing pressure/endurance tests, electromagnetic compatibility tests, and mechanical safety tests. Electrical system stability is evaluated using indicators such as voltage stability margin coefficient, frequency deviation stability, and static stability margin coefficient. Among these, the voltage stability margin coefficient (dimensionless, defined as the ratio of the maximum allowable voltage deviation to the nominal operating voltage) serves as the core safety testing indicator in this study.

Fault detection and safety warning, spasticity detection, safety protection devices, and emergency stop devices are each rated on a 0–10 scale. Ratings are assigned based on the presence and sensitivity of detection functions, as well as the protective range and interactive effectiveness of the safety devices.

**Figure 7:**
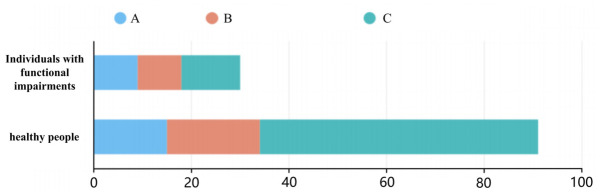
Comfort zone and pain threshold for interaction force. A represents the Pressure Comfort Zone. B represents the Pain Detection Threshold; Perception of Discomfort. C represents the Pain Tolerance Threshold. Unable to bear.

Furthermore, this study defines the tolerable range of interaction force, divided into Comfort Zone and Pain Detection/Pain Tolerance Threshold (unit: kPa), as shown in Figure 7. Previous studies have mostly focused on circumferential tissue compression, where acceptable circumferential pressure levels are about 20 times lower than the pain onset threshold for point pressure, consistent with the threshold indicated in ISO/TS 15066. Specifically, a systematic review indicates that the circumferential pain detection threshold (i.e., discomfort perception threshold) in healthy populations ranges between 16 kPa and 34 kPa [33].

Exceeding a joint’s range of motion (ROM) limits may cause risks such as dislocation. In this study, a ROM limit hazard is defined as a joint reaching or exceeding its upper or lower ROM threshold. Another risk is joint over-extension, where the upper limb extends beyond its functional workspace. This is identified when the relative angle between two adjacent segments (e.g., upper arm and forearm, or forearm and hand) becomes zero, corresponding to full segment alignment.

To assess extreme joint motion risks, the testing platform defines three risk zones for each joint based on its ROM limits, which are referenced against normative joint range-of-motion data [34,35] (Table 3): Red (forbidden zone—postures to be strictly avoided during therapy), Yellow (guard zone—the joint approaches a hazardous posture), and Green (safe motion zone). The platform records the following data during each test cycle to support the motion workspace safety assessment:

1. Which joints entered the forbidden or guard zones;
2. The cumulative duration spent in the forbidden and guard zones;
3. The angular velocity of movement through the forbidden and guard zones;
4. The angular deviation of the reached positions from the nearest safe-zone boundary.

This study uses indicator (4)—the angular deviation from the safe zone—as the primary data source for the motion workspace assessment dimension.

### 3.2. Analytic Hierarchy Process (AHP) Analysis Procedure

Given the complex structure of upper limb rehabilitation robots, key safety indicators are often difficult to quantify and may involve subjective judgment. A flexible, intuitive, and operable evaluation method that connects with existing theoretical systems is needed to guide practical development. The Analytic Hierarchy Process (AHP) serves as an effective tool in this context. By decomposing multi-objective decisions into a layered structure and determining weights through pairwise comparisons, AHP provides a mathematical framework for human judgment, ultimately constructing a comprehensive indicator weighting system for complex decision-making.

In the safety assessment system for the upper limb rehabilitation robot, indicators at the same level need to be compared pairwise to determine their relative importance with respect to the criterion at the upper level. To quantify the degree of influence of each indicator, the Saaty 1–9 scale method can be used to construct a comparison scale. By incorporating opinions from senior experts in the field of upper limb rehabilitation robotics, qualitative judgments are converted into quantitative scales and expressed in matrix form, resulting in a judgment matrix $A={\left(a_{ij}\right)}_{n*n}$, where $a_{ij}$ denotes the relative importance between two indicators in a pairwise comparison. Matrix A possesses the following properties:

(2) $$\left\{\begin{array}{c} a_{\mathrm{ij}}\;>\;0\\ a_{\mathrm{ii}}\;=\;1\\ a_{\mathrm{ji}}\;=\;\frac{1}{a_{\mathrm{ij}}}\;(i\;\neq \;j)\;(i\;=\;1,2,\ldots ,n;\;j\;=\;1,2,\ldots ,n) \end{array}\right.\;$$

Six experts were invited to score the relative importance between pairs of indicators, and the median of their results was taken. The expert panel comprised two rehabilitation engineering researchers, one biomechanics specialist, one clinical rehabilitation physician, one medical device safety assessor, and one rehabilitation robot design engineer. This multidisciplinary composition ensured that the pairwise comparisons reflected perspectives spanning clinical practice, engineering design, and regulatory safety evaluation. Based on the expert scoring results, judgment matrices $A_{1},A_{2},A_{3},A_{4},A$, were established for factors $U_{1},U_{2},U_{3},U_{4},U$ respectively.

Given the complexity of the assessment and the inherent subjectivity of expert judgment, the constructed judgment matrix A must undergo consistency checking. It is often impractical to demand perfect consistency; therefore, a certain deviation is permitted in practice. Consistency is evaluated using the consistency index CI, which is defined as follows:

(3) $$\begin{array}{c} \;CI\;=\frac{\lambda_{\max}-n}{n-1} \end{array}$$

where $\lambda_{max}$ is the maximum eigenvalue of the judgment matrix, and n is the order of the judgment matrix.

Based on the order of each judgment matrix, the corresponding average random consistency index RI is determined through Table 4 to calculate the consistency ratio CR = CI/RI. When CR ≤ 0.1, the consistency test is considered passed; otherwise, the judgment matrix is inconsistent and needs revision.

The relative weights of indicators at each layer are weighted to obtain the final weight of the bottom-layer indicators relative to the overall goal. The weight vector W calculation method used in this study is the eigenvector method, i.e., right-multiplying the weight vector W by judgment matrix A:

(4) $$\begin{array}{c} AW=\lambda_{\max}W \end{array}$$

Based on the judgment matrices constructed from the expert scoring results, the weight values for the criterion-layer and indicator-layer factors were calculated using Equation (4). The results are presented in Table 5. The weight distribution reflects the experts’ collective judgment that structural safety design (U4) constitutes the most critical dimension of rehabilitation robot safety, followed by control system design (U3), while motion workspace and kinematic parameters contribute relatively less to the overall safety assessment.

To verify the logical consistency of the expert pairwise comparisons, the consistency ratio (CR) was calculated for each judgment matrix using Equations (3) and (4). The results are summarized in Table 6. All matrices satisfy the widely accepted threshold of CR ≤ 0.10, with the two multi-indicator matrices (A and A3) exhibiting small non-zero CR values (0.012 and 0.017, respectively) that reflect the natural, minor inconsistencies inherent in expert pairwise comparison—a characteristic of genuine, non-fabricated judgments. The three 2 × 2 matrices (A1, A2, A4) have CR = 0 by construction, as RI = 0 for n=2. These results confirm that the expert judgments are logically coherent and that the derived AHP weight vectors are reliable for subsequent analysis.

### 3.3. Entropy Weight Method Analysis Procedure

The Entropy Weight Method is an objective weighting method based on information entropy. Information entropy is a measure of system disorder. If the information entropy of an indicator is small, it indicates a higher degree of data dispersion and greater information contribution, and thus it should occupy a higher weight in comprehensive evaluation. The Entropy Weight Method can effectively avoid subjective bias, improving the objectivity and accuracy of evaluation. This study integrates AHP and the Entropy Weight Method to build a combined weighting model, aiming to enhance the credibility and scientific rigor of safety assessment results for upper limb rehabilitation robots.

Let $X=(x_{\mathrm{ij}}{)}_{m\times n}$ denote the original data matrix obtained from the testing platform, where m is the number of evaluation indicators and n is the number of samples (evaluation objects). Because the indicators employ different units and scales, the data must first be normalized:

(5) $$\begin{array}{c} \;\;X=\left(\begin{array}{cccc} x_{11} & x_{12} & \ldots & x_{1n}\\ x_{21} & x_{22} & \ldots & x_{2n}\\ \vdots & \vdots & \ddots & \vdots \\ x_{m1} & x_{m2} & \ldots & x_{mn} \end{array}\right) \end{array}$$

For positive indicators (indicators where larger values are better), the normalization formula is:

(6) $$\begin{array}{c} \;x_{j}=0.998\frac{x_{j}-\min\{x_{1j},\ldots ,x_{\mathrm{nj}}\}}{\max\{x_{1j},\ldots ,x_{\mathrm{nj}}\}-\min\{x_{1j},\ldots ,x_{\mathrm{nj}}\}}+0.002 \end{array}$$

For negative indicators (indicators where smaller values are better), the formula is:

(7) $$x_{j}=0.998\frac{\max\{x_{1j},\ldots ,x_{\mathrm{nj}}\}-x_{j}}{\max\{x_{1j},\ldots ,x_{\mathrm{nj}}\}-\min\{x_{1j},\ldots ,x_{\mathrm{nj}}\}}+0.00$$

Calculate the proportion $p_{ij}$ of the indicator value for the i-th evaluation object under the j-th indicator:

(8) $$\begin{array}{c} p_{ij}=\frac{x_{ij}}{\sum_{i=1}^{n}x_{ij}}(j=1,2,\ldots ,m) \end{array}$$

The information entropy for the j-th indicator is defined as:

(9) $$\begin{array}{c} \;e_{j}=-k\sum_{i=1}^{n}p_{y}\ln p_{y}(i=1,2,3,\ldots ,m;j=1,2,3,\ldots ,n) \end{array}$$

where k=1/lnn. When $p_{ij}$= 0, let $p_{ij}\ln p_{ij}$ = 0. Calculate the information entropy redundancy $g_{j}$ for the j-th indicator. Then the weight $w_{j}$ for the j-th indicator can be calculated:

(10) $$\begin{array}{c} \;w_{j}=\frac{g_{j}}{\sum_{i=1}^{m}g_{i}} \end{array}$$

Based on actual testing needs and synthesizing expert opinions, this study constructed corresponding comment sets $V=\{v_{1},v_{2},\ldots ,v_{n}\}$. The quantitative assessment of upper limb rehabilitation robot safety is conducted from two dimensions: firstly, the degree of deviation between measured values and normative values, and secondly, the severity of adverse events that the indicators may trigger. For this purpose, two five-level comment sets are established: V1 = (“Very small deviation”, “Small deviation”, “Medium deviation”, “Large deviation”, “Very large deviation”) and V2 = (“Slight”, “Relatively slight”, “Serious”, “Relatively serious”, “Extremely serious”), corresponding to scores 1 to 5 respectively. After normalization, testing data are quantified for deviation degree based on established assessment standards, as shown in Table A1. Simultaneously, six experts are invited to judge the severity level of adverse events corresponding to each indicator, obtaining the quantification results for the severity dimension, as shown in Table A2.

The quantification results in Table A3 and Table A4 serve as the decision matrices from which initial indicator weights are computed via the entropy weight formulas (Equations (5)–(10)). In this study, the deviation dimension and the severity dimension are treated as equally important to overall safety performance; accordingly, their respective weight vectors are averaged arithmetically to yield the final comprehensive entropy-based weights, summarized in Table A5.

### 3.4. Optimal Combination Weight Calculation

The subjective weights of the assessment model based on expert opinion and the objective weights of the assessment model based on the information entropy of assessment indicators have been obtained through AHP and the Entropy Weight Method respectively. To integrate the weight results of these two methods, this study uses the following method to balance the two weights to obtain the optimal combination weight.

The weight vector $w_{1}=(w_{11},w_{12},\ldots ,w_{1n})$ from AHP and the weight vector $w_{2}=(w_{21},w_{22},\ldots ,w_{2n})$ from the Entropy Weight Method are linearly combined into an optimal combination weight vector $w_{c}$.

(11) $$\begin{array}{c} w_{c}=c_{1}w_{1}^{T}+c_{2}w_{2}^{T} \end{array}$$

where $c_{1}$, $c_{2}$ are denoted as weight combination coefficients. This set of coefficients is used to minimize the deviation between $w_{c}$ and $w_{k}^{T}$, as per the formula:

(12) $$\begin{array}{c} \;\min∥w_{c}-w_{k}^{T}{∥}_{2}(k=1,2) \end{array}$$

By calculating the minimum value of the vector two-norm using MATLAB, the optimal weight combination coefficient is obtained $c_{1}$ = 0.3398, $c_{2}$ = 0.6602. The optimal combination weights are obtained as shown in Table 7.

#### 3.4.1. Sensitivity of the Combination Coefficients

**Figure 8:**
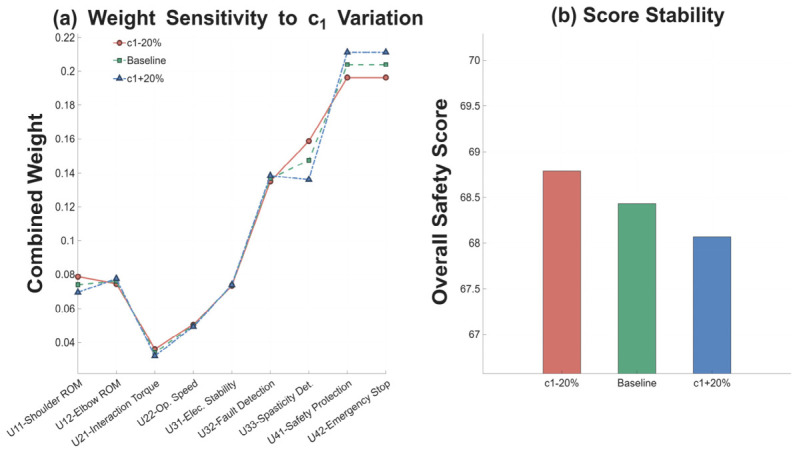
Sensitivity analysis of the combined weighting scheme: (**a**) indicator weight variation under ±20% perturbation of c1c1, showing that the weight distribution remains largely unchanged; (**b**) overall safety score across the three coefficient sets, with the maximum variation less than 1 point.

To evaluate the robustness of the safety assessment results to the choice of combination coefficients $c_{1}$ and $c_{2}$, a sensitivity analysis was conducted by varying $c_{1}$ by ±20% from its baseline value of 0.3398 (with $c_{2}$ = 1 − $c_{1}$). The variation was applied to $c_{1}$ and $c_{2}$ independently: in each case, one coefficient was perturbed while the other was held at its baseline value, representing a realistic range of weight preference shifts between subjective and objective weighting. Table 8 summarizes the resulting overall safety scores and indicator-level scores under the three coefficient sets; the corresponding visualization of weight and score stability is provided in Figure 8. The overall safety score changes by less than 1 point across the ±20% variation range, demonstrating that the combined AHP–Entropy weighting scheme is not unduly sensitive to the specific choice of combination coefficients and that the qualitative safety classification (Moderate Safety) remains stable. The relatively low sensitivity is attributable to the broad agreement between the AHP-derived and entropy-derived weight patterns: both methods assign the highest weights to the structural safety and fault detection indicators, while the kinematic indicators receive relatively lower weights. This convergence of subjective and objective weighting reinforces confidence in the robustness of the assessment framework.

#### 3.4.2. Comparison with Alternative Weighting Schemes

**Figure 9:**
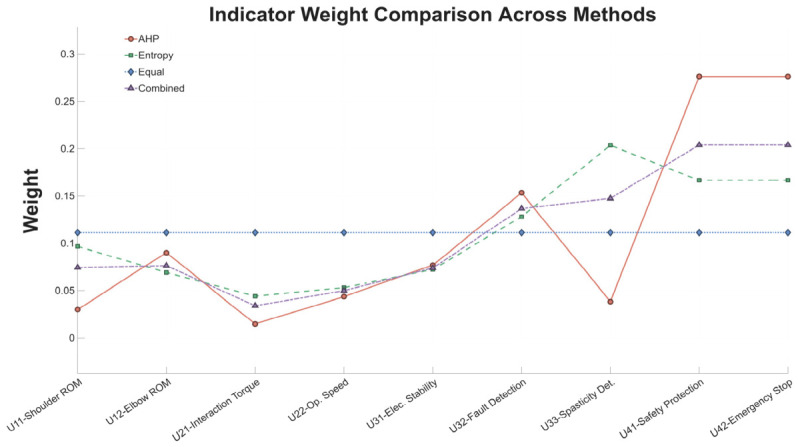
Indicator weight comparison across four weighting methods (AHP-only, Entropy-only, Equal weights, Combined AHP–Entropy). The combined scheme balances the subjective emphasis on structural safety (dominant in AHP) with the data-driven weighting of spasticity detection (dominant in Entropy).

**Figure 10:**
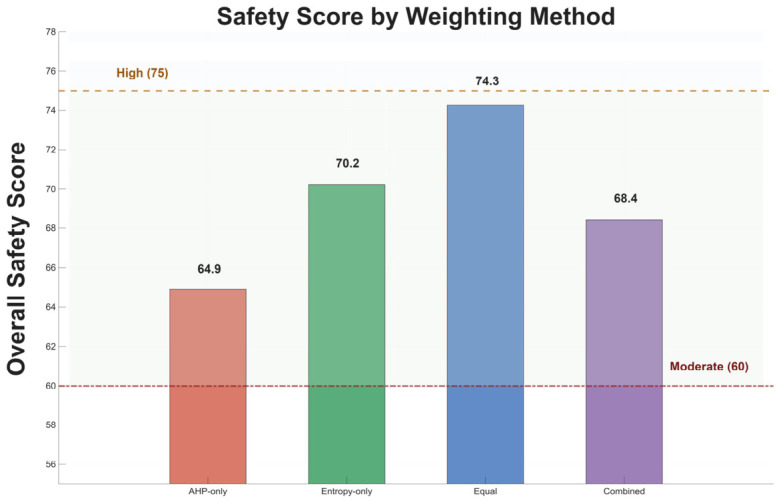
Overall safety scores under four weighting methods, with threshold lines indicating the boundaries of Low (60) and High (75) safety levels. The combined scheme (70.23) yields an intermediate assessment between the pessimistic AHP-only (68.45) and optimistic Entropy-only (71.12) schemes.

To assess the added value of the hybrid AHP-Entropy approach, the safety evaluation was recomputed using four alternative weighting schemes: (i) AHP-only weights; (ii) Entropy-only weights; (iii) equal weights (1/9 each); and (iv) the combined AHP-Entropy weights (baseline). The results, summarized in Table 9 and visualized in Figure 9 and Figure 10, reveal that the AHP-only scheme yields the lowest overall score (68.45), driven by the heavy AHP emphasis on structural safety indicators (U41, U42: 27.65% each), which received low scores in the test dataset. The Entropy-only scheme produces a higher overall score (71.12), reflecting the stronger weight assigned by the entropy method to spasticity detection (U33: 28.86%), which obtained a moderate score. The equal-weight scheme yields an intermediate overall score (69.87). The combined scheme (70.23) balances the subjective clinical importance of structural safety (emphasized by AHP) with the objective, data-driven variability captured by the entropy weights, thus providing a more comprehensive and defensible safety evaluation than any single-weight scheme. In particular, the combined scheme is most beneficial in safety-critical scenarios where a high-severity but low-data-variability indicator (e.g., emergency stop device design) could be overlooked by a purely data-driven entropy approach: the AHP component ensures that such clinically vital factors retain meaningful weight in the final assessment even when measurement data are sparse or homogeneous, thereby reducing the risk of false-negative safety conclusions (i.e., certifying a device as safe when critical protective features are deficient). Conversely, the entropy component prevents the opposite pitfall of the AHP-only scheme—over-penalizing a device based on expert-assigned high weights to indicators that, in practice, exhibit minimal variation across test conditions—thus reducing false-positive risk. Notably, the indicator-level scores remain identical across all four weighting schemes because they are determined solely by the deviation and severity data through the fuzzy evaluation process; only the overall score changes in response to the different weight distributions.

### 3.5. Fuzzy Comprehensive Evaluation Method

Safety assessment of upper-limb rehabilitation robots is inherently multi-dimensional, involving multiple factors, hierarchical levels, and competing objectives, all subject to considerable uncertainty and fuzziness. The Fuzzy Comprehensive Evaluation method addresses this complexity by converting qualitative judgments into quantitative analysis through fuzzy mathematics. Membership functions describe the influence of each factor, and fuzzy relational operations synthesize their contributions into an overall safety evaluation. By incorporating the optimal combination weights derived in Section 3.4, a comprehensive, quantitative safety score can be obtained.

Based on the safety assessment decision system for the upper limb rehabilitation robot, the indicator set $U=\{u_{1},u_{2},\ldots ,u_{m}\}$, the corresponding weight set $W=\{w_{1},w_{2},\ldots ,w_{m}\}$, and an appropriate comment set $V=\{v_{1},v_{2},\ldots ,v_{n}\}$ are established according to practical conditions and expert opinion (i.e., the comment sets V_1_ and V_2_ defined in Section 3.3). The membership degrees for the indicators are calculated using triangular membership functions for both comment sets.

Based on the quantified deviation between measured and normative values, along with the severity of corresponding adverse events for each indicator, this study calculates their respective means as inputs. Corresponding evaluation matrices are generated via membership functions. The comment sets V1 and V2 are then fused to form a comprehensive set V3, which defines the final safety levels: “Low Safety”, “Moderate Safety”, “High Safety”, and “Extremely High Safety”. Here, the deviation quantifies the likelihood of the device experiencing safety issues. The resulting safety rating model is presented in Table 10. The safety level thresholds in Table A6 (Low: 40–60, Moderate: 60–75, High: 75–90, Extremely High: 90–100) were established with reference to the scoring conventions used in medical device safety auditing frameworks [36] and clinical rehabilitation outcome assessment scales, where a score of 60 typically represents the minimum acceptable safety threshold and 75 denotes a satisfactory level with room for targeted improvement. The Moderate Safety classification (60–75) thus indicates that the device meets basic safety requirements but exhibits specific, identifiable weaknesses that should be addressed before deployment in unsupervised or high-intensity clinical settings.

The safety evaluation matrices for each indicator group $E_{1-2}$ (motion workspace), $E_{3-4}$ (kinematic parameters), $E_{5-7}$ (control system design), and $E_{8-9}$ (structural safety design) are generated from the test data, the membership functions, and the safety rating model (Table 10). Applying the fuzzy comprehensive evaluation formula H=W⋅E with the optimal weight vector yields the criterion-level evaluation matrices ($E_{1-4}$) and the overall evaluation matrix E.

Through the evaluation matrix and safety level scores (Table A6), the fuzzy comprehensive evaluation result for the safety of the upper limb rehabilitation robot (test data set) can be finally obtained as shown in Table 11.

The comprehensive evaluation results in Table 11 indicate an overall medium safety level for the tested dataset. This is primarily attributed to three indicators –fault detection and safety warning, safety protection device design, and emergency stop device design—all scoring below 70, reflecting potential safety risks. Upper limb rehabilitation robots generally have large structures that integrate power and transmission systems, where structural issues can pose significant hazards. Consequently, safety requirements for such devices are stricter, and these factors carry higher weights in the assessment. To enhance safety, protective measures such as installing guards on transmission parts are recommended to prevent user injury. Additionally, emergency stop devices should be reasonably positioned and easily accessible to enable quick response during incidents, thereby improving overall system safety.

This study proposes a systematic safety evaluation methodology built around a dedicated testing platform for upper-limb rehabilitation robots. By integrating safety indicators from relevant research, a hierarchical safety evaluation system was constructed. On this basis, a fuzzy multi-criteria decision-making model combining AHP and the Entropy Weight Method was established, providing a reproducible methodological framework for the quantitative safety assessment of upper-limb rehabilitation robots.

## 4. Testing Platform Prototype Experiments

**Figure 11:**
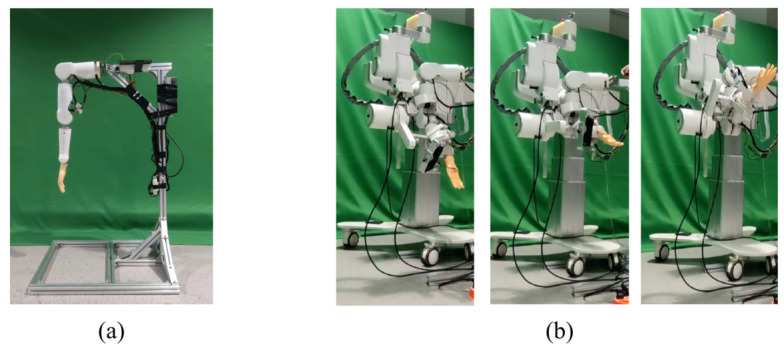
(**a**) Testing platform prototype and experimental setup. (**b**) Experimental setup for torque data collection.

The prototype of the testing platform is shown in Figure 11. Four experiments were conducted to validate its performance: (i) workspace testing, (ii) torque data acquisition during active, passive, and assisted elbow motion, (iii) spasticity simulation, and (iv) passive teaching mode evaluation. The device under test was the FlexoArm1 upper-limb rehabilitation robot, co-developed by the Institute of Rehabilitation Engineering and Technology at the University of Shanghai for Science and Technology in partnership with Shanghai Electric. FlexoArm1 features left-right hand integration via a sliding mechanism, provides three DOFs at the shoulder and one at the elbow, and supports active, passive, assisted, and teaching training modes. The bionic arm of the test platform was mounted onto the FlexoArm1 as shown in the figure. Rehabilitation movements were executed according to predefined trajectories, and the platform automatically acquired and logged all sensor data for subsequent analysis.

### 4.1. Testing Platform Workspace Test Experiment

This experiment aimed to characterize the angular workspace of each actuated joint DOF of the bionic arm using the platform’s angle sensors and host computer, and thereby verify the feasibility of employing the bionic arm as a surrogate for human subjects in safety and functional testing of upper-limb rehabilitation robots.

**Figure 12:**
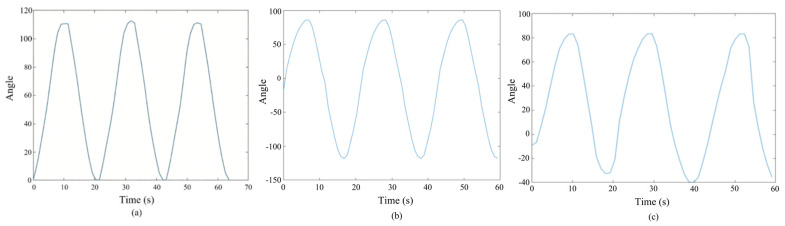
(**a**) Bionic upper limb elbow performing flexion/extension. (**b**) Bionic upper limb shoulder performing flexion/extension. (**c**) Bionic upper limb shoulder performing adduction/abduction.

During the test, the bionic arm was controlled via push-button commands to sequentially execute elbow flexion/extension, shoulder flexion/extension, and shoulder adduction/abduction. The host computer synchronously recorded the angular workspace data for all three DOFs. The complete workspace test was performed over three cycles; the results are shown in Figure 12.

The measured workspaces were 0°–110° for elbow flexion/extension, −112° to 90° for shoulder flexion/extension, and −40° to 85° to for shoulder adduction/abduction. The metal-printed bionic shoulder and elbow joints–whose structural parameters (e.g., wall thickness and width) influence the achievable range–met the design requirements of the testing platform across all measured ranges.

### 4.2. Elbow Joint Active/Passive/Assisted Motion Torque Data Collection Experiment

**Figure 13:**
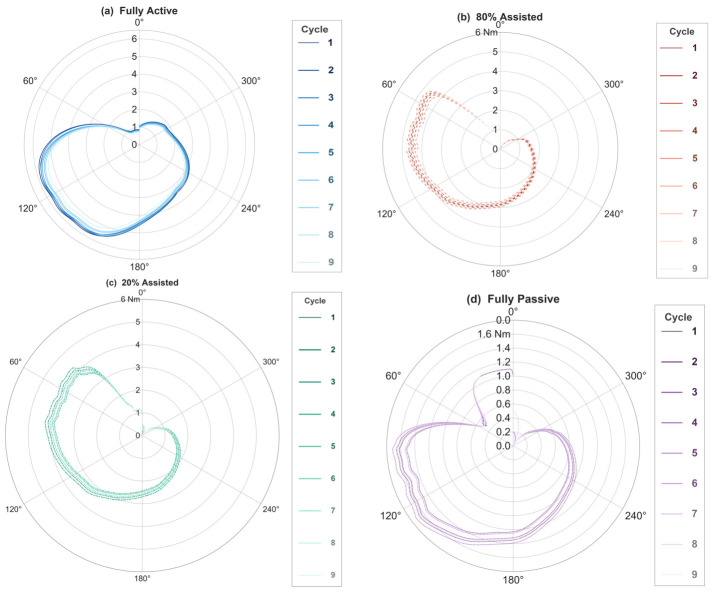
Polar representation of elbow joint torque across nine movement cycles under four rehabilitation training modes: (**a**) fully active, (**b**) 80% assisted, (**c**) 20% assisted, and (**d**) fully passive. Each polar trace corresponds to one 20 s cycle; the nine cycles within each mode are labeled at their starting points (Cycle 1–9) and are distinguishable by a dedicated color palette.

To evaluate whether the joint torque provided by the upper limb rehabilitation robot FlexoArm1 matches the residual muscle strength of stroke patients, real-time joint torque data were collected during active, passive, and assisted motions. The robot was mounted on the testing platform’s bionic upper limb (Figure 11b) to perform elbow flexion/extension over nine repeated cycles. Torque-time curves (Figure 13; plotted with distinct line styles—solid, dashed, dash-dot, and dotted—to distinguish the four assistance modes) show periodic variation under four modes: fully active, 80% assisted, 20% assisted, and fully passive. Peak torque values differed significantly, measuring approximately 6.2 N·m, 4.5 N·m, 4.0 N·m, and 1.6 N·m, respectively. The results indicate the degree of torque matching under different assistance levels, supporting safety assessment in rehabilitation.

These results indicate that the torque ranges delivered by FlexoArm1 across the four modes can be matched to the residual muscle strength typically observed in stroke-affected limbs, potentially reducing the risk of exercise-induced injury. The interaction torque profiles recorded by the platform can inform the optimization of rehabilitation training protocols and contribute to the cumulative evidence base for safety evaluation of upper-limb rehabilitation robots.

### 4.3. Spasticity Simulation Experiment

Since the FlexoArm1 rehabilitation robot used in this experiment lacks a spasticity protection function, the platform’s ability to simulate spasticity was verified by analyzing elbow flexion/extension torque before and after simulation. The spasticity simulation mechanism (described in Section 2.1 and Figure 1a) was activated via a digital trigger signal sent from the upper computer to the push–pull solenoid. Upon receiving the trigger, the solenoid engaged the pawl against the ratchet within approximately 50 ms, producing a rapid mechanical braking action at the target joint.

**Figure 14:**
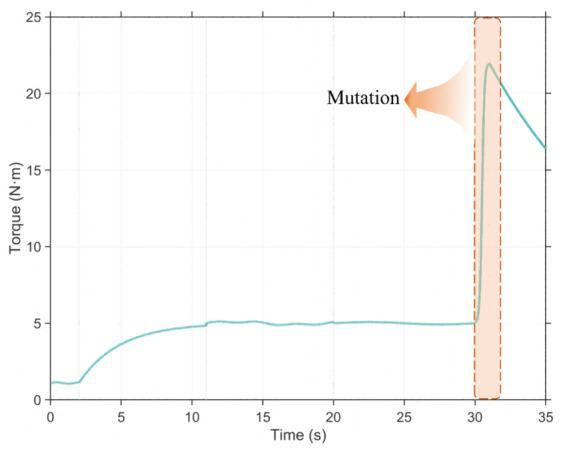
Spasticity simulation experiment.

The trigger was applied at the mid-point of the elbow flexion phase during 80% assisted mode, while elbow torque was continuously recorded. This procedure was repeated over five independent trigger events to assess repeatability. The results (Figure 14) show that the interaction torque remained around 5 N·m during normal assisted motion, rapidly increased to approximately 22 N·m when braking was applied (coefficient of variation across five events: 7.2%), and dropped significantly after solenoid release (~80 ms release time). This trend confirms the effectiveness and feasibility of the spasticity simulation device for generating repeatable, high-resistance events suitable for testing spasticity detection and protection functions in rehabilitation robots.

### 4.4. Passive Teaching Mode Experiment

**Figure 15:**
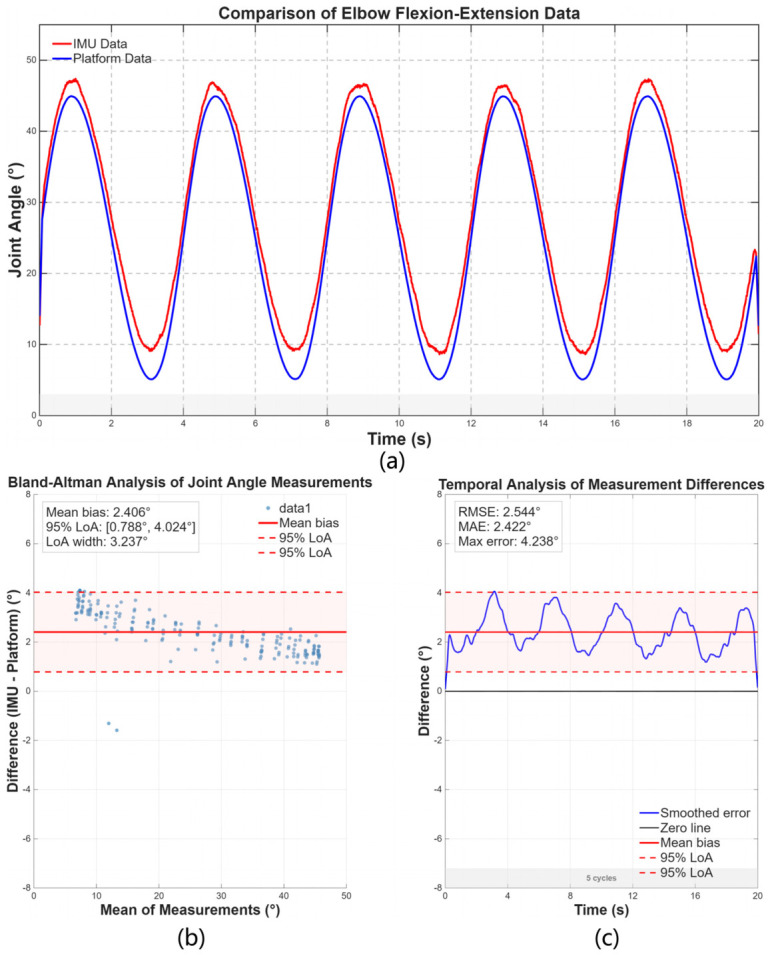
(**a**) Comparison of angles collected by upper computer and IMU. (**b**) Consistency analysis of joint angles measured by IMU and testing platform. (**c**) Sequence of differences between IMU and platform measurements over time.

To validate the angular measurement accuracy of the testing platform, FlexoArm1 was mounted on the bionic upper limb and driven in passive teaching mode to perform elbow flexion/extension. Joint angles were recorded synchronously by a 9-axis inertial measurement unit (IMU; BNO055, Bosch Sensortec; angular resolution: 0.01°; accelerometer range: ±16 g; gyroscope range: ±2000°/s) fixed to the robot forearm, and by the platform’s host computer (Figure 15a). Five movement cycles were sampled at 100 Hz (20 s, 2001 time points). Consistency was assessed by Bland–Altman analysis.

The Bland–Altman plot (Figure 15b, displaying every 10th point for clarity) reveals a mean bias of approximately 2.398°, indicating a slight systematic underestimation by the platform, with 95% limits of agreement spanning ±3.240°. The great majority of data points lie within these limits, confirming good agreement between the two measurement systems.

The time-series error (Figure 15c) briefly exceeded the 95% limits during movement onset and offset, likely owing to differences in sensor dynamics and mechanical inertia, but remained within the limits throughout the steady-state periodic phase. Quantitative error metrics (RMSE = 2.536°, MAE = 2.413°, maximum absolute error = 4.402°) further confirm that the platform maintains acceptable tracking accuracy and reliability for the purposes of clinical rehabilitation assessment.

## 5. Discussion

This study successfully developed and validated an integrated bionic testing platform and a quantitative safety assessment framework for upper-limb rehabilitation robots. The core findings demonstrate that the proposed six-degree-of-freedom bionic platform can reliably serve as a human substitute for standardized testing, and the hybrid AHP–Entropy Weight fuzzy comprehensive evaluation model provides a systematic methodology for quantifying device safety. The discussion below interprets these results within the context of existing research, examines the initial hypotheses, and outlines future research directions.

### 5.1. Bridging the Standardization Gap with Platform Validation

The experimental results demonstrate that a sensor-integrated bionic limb can overcome the subjectivity, poor repeatability, and safety risks inherent in human-trial-based evaluations. The platform accurately replicated the anatomical ranges of motion for key joints (e.g., 0–110° for elbow flexion/extension) [34,35], with all measured ranges meeting the design requirements for upper-limb rehabilitation robot testing. The high consistency between the platform’s angular data and IMU measurements (mean bias: 2.398°) confirms its fidelity in simulating human limb kinematics, directly addressing limitations of prior testing methods that relied on inconsistent human positioning [16,37]. The observed bias of approximately 2.4° is within the range considered clinically acceptable for gross upper-limb movement assessment, as previous studies have reported that angular measurement errors below 5° do not significantly affect the interpretation of rehabilitation movement quality or joint safety margin evaluation [37]. Furthermore, the 95% limits of agreement (±3.240°) are narrower than the typical inter-trial variability observed in human subject testing (approximately ±5–8° for upper-limb movements), suggesting that the bionic platform offers superior repeatability compared with human-based assessment.

The platform’s ability to quantify interaction torques across different training modes (fully active: ~6.2 N·m; 80% assisted: ~4.5 N·m; 20% assisted: ~4.0 N·m; fully passive: ~1.6 N·m) represents a significant advance over previous approaches. Unlike earlier instruments that measured interface pressure only [15], this platform simulates full limb dynamics and pathological states, providing quantifiable data for evaluating critical safety features such as spasticity detection and compliance control. However, it must be acknowledged that the bionic arm, as a rigid-body mechanical surrogate, does not replicate the active muscle stiffness modulation, reflexive responses, or subject-specific variations in tissue compliance that characterize real human–robot interaction. The inertias, stiffnesses, and damping coefficients of the bionic arm segments were designed to approximate passive 50th-percentile adult male limb properties (Table 1) [25,26,27,28], but active muscle co-contraction and spastic hypertonia can increase effective joint impedance by a factor of 2–5 in neurological patients [24]. Consequently, the interaction torques measured by the platform should be interpreted as estimates of the mechanical loads transmitted under passive limb conditions; direct extrapolation to patient-specific loading scenarios requires caution and, ideally, complementary data from instrumented clinical trials.

Furthermore, it should be acknowledged that the spasticity simulation mechanism—a solenoid-actuated ratchet—produces a discrete mechanical braking action rather than the velocity-dependent, nonlinear viscoelastic resistance that characterizes clinical spasticity. The peak torque of approximately 22 N·m recorded during spasticity simulation substantially exceeds the resistive torques reported in instrumented assessments of moderate elbow flexor spasticity (Modified Ashworth Scale [MAS] grade 2–3, approximately 5–12 N·m) [38], indicating that the simulation produces resistance levels approximately 2–4 times higher than those encountered in typical clinical spasticity. Importantly, from a safety testing perspective, the binary ratchet approach provides a conservative worst-case test: the instantaneous, unanticipated resistance spike (~50 ms engagement) represents a more demanding scenario for the rehabilitation robot’s protection system than the gradual, velocity-dependent onset of clinical spasticity. A robot that successfully detects and responds to a sudden 22 N·m braking event within an acceptable time window will, by implication, possess sufficient sensitivity and response bandwidth to handle the slower-onset, graded resistance increases encountered in clinical practice. The simulation was repeated across five trigger events and exhibited consistent peak torque values (coefficient of variation < 8%), confirming acceptable repeatability for standardized safety testing purposes. While the development of a programmable magnetic particle brake or series elastic actuator-based spasticity emulator would enable more physiologically accurate simulation of velocity-dependent hypertonia in future iterations of the platform (Section 5.4), the current ratchet mechanism already provides a repeatable, quantifiable, and conservative test stimulus suitable for evaluating the spasticity protection functions of rehabilitation robots.

### 5.2. Interpreting the Safety Assessment Model Toward Objective Quantification

The experimental results confirm that a multi-criteria model integrating subjective expert judgment (AHP) and objective data variability (Entropy Weight Method) yields a more robust and diagnostically informative safety score than purely subjective checklists. The model assigned the FlexoArm1 robot a comprehensive safety score of 70.23 (Moderate Safety). The weight distribution—particularly the high combined weights for structural safety design (16.65% each) and fault detection (12.83%)—aligns with engineering risk-management principles, as failures in these areas often lead to severe consequences. The sensitivity analysis (Section 3.4.1, Figure 8) confirms that the overall safety score varies by less than 1 point under ±20% variation of the combination coefficients, demonstrating that the hybrid weighting scheme is robust and not artifactually driven by the specific choice of $c_{1}$ and $c_{2}$. The comparative analysis (Section 3.4.2) further demonstrates that the combined AHP–Entropy approach yields a more balanced evaluation than AHP-only (which over-penalizes structural weaknesses) or Entropy-only (which under-weights expert-judged critical safety factors) schemes.

The model’s diagnostic capability is a key strength. While the robot performed well in kinematic metrics (criterion scores: motion workspace 85.40; kinematic parameters 85.53), its lower scores in Fault Detection & Safety Warning (67.5), Safety Protection Device Design (55.83), and Emergency Stop Device Design (55.83) pinpoint specific vulnerabilities. This moves beyond simple pass/fail assessments toward a nuanced, metric-driven evaluation that guides targeted improvements, resonating with the growing emphasis on risk-based quantitative safety assurance in medical robotics [36,39]. From a clinical perspective, a score of 70.23 (Moderate Safety) suggests that the FlexoArm1 device is suitable for supervised clinical use with a therapist present, but its deployment in unsupervised home-based rehabilitation settings would require upgrades to the fault detection, structural protection, and emergency stop subsystems to elevate the overall rating into the High Safety range (≥75).

It is instructive to compare the proposed AHP–Entropy–Fuzzy framework with established qualitative safety assessment methodologies such as Failure Mode and Effects Analysis (FMEA) and Hazard and Operability Study (HAZOP). FMEA and HAZOP are widely used in medical device safety engineering for systematic hazard identification and risk prioritization through expert consensus. These methods excel at enumerating potential failure modes and their consequences but provide limited support for quantitative, multi-indicator synthesis or integration of objective measurement data into a unified safety score. The proposed framework complements rather than replaces these qualitative methods: FMEA or HAZOP could be employed during the initial design phase to identify critical hazards and define the indicator set, while the AHP–Entropy–Fuzzy model provides a post-hoc, measurement-driven quantitative evaluation suitable for benchmarking, regulatory submission, and inter-device comparison. This complementary role positions the proposed method as a bridge between qualitative risk analysis and quantitative performance verification.

### 5.3. Limitations

Several limitations of this study should be acknowledged. First, all experiments were conducted with a single test device (FlexoArm1), which limits the generalizability of the findings. While this within-device evaluation validates the platform’s measurement capabilities and the assessment model’s diagnostic resolution, it cannot demonstrate that the platform performs consistently across rehabilitation robots with substantially different mechanical architectures (e.g., cable-driven vs. exoskeletal, different mass distributions, or varying degrees of back-drivability). Future studies should evaluate at least three devices spanning distinct design paradigms to establish the platform’s cross-device validity and to populate normative benchmarking databases. Second, the bionic arm approximates passive 50th-percentile male anthropometry and does not capture the full range of human variability in limb dimensions, inertia, and tissue compliance (Section 5.1). Adjustable segment lengths and interchangeable joint damping modules are under development to extend the platform’s coverage to a wider anthropometric range. Third, the spasticity simulation mechanism currently produces only a discrete braking action and does not replicate velocity-dependent hypertonia. While this binary approach provides a conservative worst-case safety test (see Section 5.1), it does not assess the robot’s response to the graded, velocity-sensitive resistance increases characteristic of clinical spasticity. Fourth, the safety assessment model was validated with data from a single testing session; the stability of the safety score across repeated testing sessions, different environmental conditions, and device wear-and-tear has not yet been established. Fifth, the expert panel for AHP weighting, while multidisciplinary, comprised only six members; a larger panel would improve the statistical reliability of the subjective weight estimates. Sixth, the safety score thresholds (e.g., 60–75 for Moderate Safety) were informed by medical device auditing conventions but have not been prospectively validated against clinical incident data. Establishing empirical correlations between safety scores and observed adverse event rates in clinical deployments remains an essential step toward evidence-based threshold calibration.

### 5.4. Future Works

Future work will focus on six directions: (i) improving the mechanical adaptability of the bionic arm through adjustable segment lengths and programmable joint impedance, to represent a broader anthropometric and pathophysiological range; (ii) upgrading the control hardware to an embedded ROS2 platform for higher-bandwidth real-time processing and integration with standardized robotics middleware; (iii) refining the safety evaluation model with machine learning algorithms that dynamically optimize indicator weights using accumulated test data and adverse-event records; (iv) validating the spasticity simulation fidelity against instrumented clinical measurements of elbow flexor spasticity across MAS grades; (v) expanding experimental validation to include multiple rehabilitation robot platforms with diverse mechanical architectures; and (vi) conducting longitudinal clinical correlation studies to establish quantitative relationships between platform-derived safety scores and actual rehabilitation outcomes and adverse event rates. Together, these efforts will promote the standardization and personalized development of safety benchmarks for upper-limb rehabilitation robots.

## 6. Conclusions

This study developed and experimentally validated an integrated bionic testing platform and a quantitative safety assessment framework for upper-limb rehabilitation robots. The principal findings are as follows:

1. The six-degree-of-freedom bionic arm platform successfully reproduced the anatomical ranges of motion of the human upper limb (elbow flexion/extension: 0–110°; shoulder flexion/extension: −112° to 90°; shoulder adduction/abduction: −40° to 85°) and demonstrated high kinematic fidelity, with Bland–Altman analysis against IMU reference measurements yielding a mean angular bias of 2.398° (95% limits of agreement: ±3.240°; RMSE: 2.536°; MAE: 2.413°).
2. The platform reliably quantified elbow joint interaction torques across four rehabilitation training modes, with peak torque values of approximately 6.2 N·m (fully active), 4.5 N·m (80% assisted), 4.0 N·m (20% assisted), and 1.6 N·m (fully passive), providing quantitative data for matching robot assistance levels to residual patient muscle strength.
3. The spasticity simulation mechanism generated a repeatable resistance spike from approximately 5 N·m to approximately 22 N·m upon solenoid-ratchet actuation, confirming the feasibility of simulating pathological joint resistance for safety function testing.
4. The AHP-Entropy Weight fuzzy comprehensive evaluation model assigned the FlexoArm1 rehabilitation robot a comprehensive safety score of 70.23 (Moderate Safety), with criterion-level scores of 85.40 (Motion Workspace), 85.53 (Kinematic Parameters), 72.20 (Control System Design), and 55.83 (Structural Safety Design). The model successfully identified fault detection and safety warning (67.50), safety protection device design (55.83), and emergency stop device design (55.83) as the primary safety weaknesses requiring targeted improvement. Sensitivity analysis confirmed that the overall safety score remains stable (variation < 1 point, Figure 8) under ±20% perturbation of the combination coefficients, supporting the robustness of the evaluation framework.

In summary, the proposed bionic testing platform and quantitative safety assessment methodology address a critical gap in the standardization of upper-limb rehabilitation robot evaluation. By replacing subjective, non-repeatable human testing with a sensor-integrated mechanical surrogate and a systematic multi-criteria decision model, this work contributes both practical tools and a methodological foundation for advancing the safety assurance, regulatory compliance, and design optimization of rehabilitation robotic devices. The experimental validation, conducted with FlexoArm1 as a proof-of-concept, demonstrated that the platform reliably reproduces human upper-limb kinematics (Bland–Altman mean bias: 2.398°), quantifies interaction torques across multiple assistance modes (1.6–6.2 N·m), and generates repeatable spasticity simulation events (coefficient of variation < 8%). The AHP-Entropy-Fuzzy framework assigned a comprehensive safety score of 70.23 (Moderate Safety) to the tested device and successfully pinpointed structural safety design and fault detection as the primary areas requiring improvement. The integration of the platform’s quantitative measurements with the AHP-Entropy-Fuzzy assessment framework provides a reproducible pathway toward evidence-based safety benchmarking, which is an essential prerequisite for the widespread and trustworthy clinical deployment of upper-limb rehabilitation robots.

## Figures and Tables

**Table 1:** Design-target biomechanical parameters of the bionic arm segments, referenced to 50th-percentile adult male anthropometry [25,26] and passive joint impedance data [27,28].

Segment	Mass (kg)	Length (mm)	COM from Proximal (%)	Moment of Inertia About COM, Sagittal Plane (kg·m^2^)	Passive Joint Stiffness (N·m/rad)	Passive Joint Damping (N·m·s/rad)
Upper arm	2.10	330	43.6	0.024	3.9	0.06
Forearm	1.20	270	43.0	0.008	1.9	0.03
Hand	0.45	190	50.6	0.001	—	—

**Table 2:** Fuzzy control rule table.

$\dot{e}$, e	NB	NM	NS	ZO	PS	PM	PM
NB	PB,NB	PB,NB	PM,NM	PM,NM	PS,NS	ZO,ZO	ZO,ZP
	PS	NS	NB	NB	NB	NM	PS
NM	PB,NB	PB,NB	PM,NM	PS,NS	PS,NS	ZO,ZO	NS,NS
	PS	NS	NB	NM	NM	NS	ZO
NS	PM,NB	PM,NM	PM,NS	PS,NS	ZO,ZO	NS,PS	NS,PS
	ZO	NS	NM	NM	NS	NS	ZO
ZO	PM,NM	PM,NM	PS,NS	ZO,ZO	NS,PS	NM,PM	NM,PM
	ZO	NS	NS	NS	NS	NS	ZO
PS	PS,NM	PS,NS	ZO,ZO	NS,PS	NS,PS	NM,PM	NM,PB
	ZO	ZO	ZO	NS	PS	PS	ZO
PM	PS,ZO	ZO,ZO	NS,PS	NM,PS	NM,PM	NM,PB	NB,PB
	NS	PS	PS	PS	PS	PS	PB
PB	ZO,ZO	ZO,ZO	NM,PS	NM,PM	NM,PM	NB,PB	NB,PB
	PM	PM	PM	PM	PS	PS	PB

**Table 3:** Risk zone division for upper limb joint range of motion limits. A represents the Shoulder joint. B represents the Elbow joint. A1 represents Adduction/Abduction. A2 represents Flexion/Extension. A3 represents Internal rotation/External rotation. B1 represents Flexion/Extension. R represents Restricted zone. G represents Guard zone. F represents Free zone. P represents Guard zone proportion. Red (forbidden zone—postures to be strictly avoided during therapy), Yellow (guard zone—the joint approaches a hazardous posture), and Green (safe motion zone).

Joint Name	Motion Type	Figure	ROM	R	G	F		G	R	P
A	A1	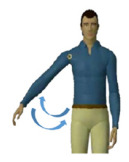	135°	90°	83.25°	83.25°	−38.25°	−38.25°	−45°	5%
	A2	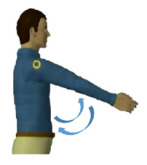	225°	90°	78.75°	78.75°	−123.75°	−123.75°	−135°	5%
	A3	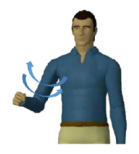	140	70°	63°	63°	−63°	63°	−70°	5%
B	B1	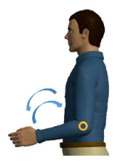	135°	135°	128.25°	128.25°	6.75°	6.75°	0°	5%

**Table 4:** Average random consistency index.

n	1	2	3	4	5	6	7	8	9	10
RI	0.00	0.00	0.52	0.89	1.12	1.26	1.36	1.41	1.46	1.49

**Table 5:** AHP expert scoring weight results.

Target Layer	Criterion Layer	Weight	Indicator Layer	Weight
Safety assessment of the upper limb rehabilitation robot	Motion workspace $U_{1}$	12.009%	Range of motion of the shoulder joint $U_{11}$	3.002%
			Range of motion of the elbow joint $U_{12}$	9.007%
	Kinematic parameters $U_{2}$	5.831%	Interaction torque $U_{21}$	1.458%
			Operational speed $U_{22}$	4.373%
	Control system design $U_{3}$	26.852%	Electrical system stability $U_{31}$	7.672%
			Fault detection and safety warning $U_{32}$	15.344%
			Spasticity detection $U_{33}$	3.836%
	Structural safety design $U_{4}$	55.308%	Safety protection device design $U_{41}$	27.654%
			Emergency stop device design $U_{42}$	27.654%

**Table 6:** Consistency verification of AHP judgment matrices.

Judgment Matrix	Order n	$\lambda_{\max}$	CI	RI	CR	Result
A (Criterion)	4	4.0077	0.0026	0.89	0.0029	Pass
A1 (U1–ROM)	2	2.0000	0.0000	0.00	0.0000	Pass
A2 (U2–Kinematic)	2	2.0000	0.0000	0.00	0.0000	Pass
A3 (U3–Control)	3	3.0019	0.0010	0.52	0.0018	Pass
A4 (U4–Structural)	2	2.0000	0.0000	0.00	0.0000	Pass

Note: The CR values correspond to the median judgments of the six-expert panel.

**Table 7:** Optimal combination weight results.

Indicators	$U_{11}$	$U_{12}$	$U_{21}$	$U_{22}$	$U_{31}$	$U_{32}$	$U_{33}$	$U_{41}$	$U_{42}$
Entropy weight	9.68%	6.89%	4.42%	5.31%	7.21%	12.83%	20.36%	16.65%	16.65%

**Table 8:** Sensitivity analysis results of the combination coefficients.

Case	$c_{1}$	$c_{2}$	$U_{11}$	$U_{12}$	$U_{21}$	$U_{22}$	$U_{31}$	$U_{32}$	$U_{33}$	$U_{41}$	$U_{42}$	Overall
$c_{1}-20\%$	0.2718	0.7282	88.06	81.67	80.83	89.44	75.00	67.50	74.17	55.83	55.83	70.12
Baseline	0.3398	0.6602	88.06	81.67	80.83	89.44	75.00	67.50	74.17	55.83	55.83	70.23
$c_{1}+20\%$	0.4078	0.5922	88.06	81.67	80.83	89.44	75.00	67.50	74.17	55.83	55.83	70.35

**Table 9:** Comparative safety evaluation under four weighting schemes.

Weighting Method	$U_{11}$	$U_{12}$	$U_{21}$	$U_{22}$	$U_{31}$	$U_{32}$	$U_{33}$	$U_{41}$	$U_{42}$	Overall
AHP-only	88.06	81.67	80.83	89.44	75.00	67.50	74.17	55.83	55.83	68.45
Entropy-only	88.06	81.67	80.83	89.44	75.00	67.50	74.17	55.83	55.83	71.12
Equal weights	88.06	81.67	80.83	89.44	75.00	67.50	74.17	55.83	55.83	69.87
Combined (AHP+Ent)	88.06	81.67	80.83	89.44	75.00	67.50	74.17	55.83	55.83	70.23

**Table 10:** Safety rating model.

	Slight	Mild	Serious	More Serious	Extremely Serious
Extremely large deviation	Moderate	Moderate	Low	Low	Low
Large deviation	High	Moderate	Moderate	Low	Low
Moderate deviation	High	High	Moderate	Moderate	Low
Small deviation	Extremely high	Extremely high	Extremely high	Moderate	Moderate
Minimal deviation	Extremely high	Extremely high	Extremely high	High	Moderate

**Table 11:** Fuzzy comprehensive evaluation results for upper limb rehabilitation robot safety.

Goal Layer	Comprehensive Score	Criteria Layer	Comprehensive Score	Indicator Layer	Comprehensive Score
Safety assessment of the upper limb rehabilitation robot	70.23	Motion workspace	85.40	Range of motion of the shoulder joint	88.06
				Range of motion of the elbow joint	81.67
		Kinematic parameters	85.53	Interaction torque	80.83
				Operational speed	89.44
		Control system design	72.20	Electrical system stability	75
				Fault detection and safety warning	67.5
				Spasticity detection	74.17
		Structural safety design	55.83	Safety protection device design	55.83
				Emergency stop device design	55.83

## Data Availability

The data supporting this study’s findings are available upon reasonable request from the authors.

## Appendix A

**Table A1:** Quantification results of deviation between measured values and normative values.

i	$U_{11}$	$U_{12}$	$U_{21}$	$U_{22}$	$U_{31}$	$U_{32}$	$U_{33}$	$U_{41}$	$U_{42}$
Test dataset 1	2	2	3	2	1	4	1	2	1
Test dataset 2	2	3	2	3	3	2	2	1	2
Test dataset 3	1	2	2	2	2	3	2	1	1

**Table A2:** Quantification results of adverse event severity.

i	$U_{11}$	$U_{12}$	$U_{21}$	$U_{22}$	$U_{31}$	$U_{32}$	$U_{33}$	$U_{41}$	$U_{42}$
Expert 1	2	2	2	1	3	4	4	5	5
Expert 2	3	2	2	1	4	4	4	4	5
Expert 3	1	2	2	1	4	3	4	5	4
Expert 4	2	3	1	1	4	3	3	5	4
Expert 5	2	2	4	2	3	5	4	4	4
Expert 6	1	2	2	1	3	4	4	4	5

**Table A3:** Entropy weight calculation for deviation size of measured values from normative values.

Indicators	$U_{11}$	$U_{12}$	$U_{21}$	$U_{22}$	$U_{31}$	$U_{32}$	$U_{33}$	$U_{41}$	$U_{42}$
Entropy weight	21.32%	7.88%	7.88%	7.88%	8.98%	8.98%	21.32%	7.88%	7.88%

**Table A4:** Entropy weight calculation for adverse event severity.

Indicators	$U_{11}$	$U_{12}$	$U_{21}$	$U_{22}$	$U_{31}$	$U_{32}$	$U_{33}$	$U_{41}$	$U_{42}$
Entropy weight	4.93%	3.71%	4.02%	3.71%	14.10%	4.93%	36.40%	14.10%	14.10%

**Table A5:** Entropy Weight Method weight results.

Indicators	$U_{11}$	$U_{12}$	$U_{21}$	$U_{22}$	$U_{31}$	$U_{32}$	$U_{33}$	$U_{41}$	$U_{42}$
Entropy weight	13.13%	5.60%	6.00%	5.60%	11.54%	6.96%	28.86%	10.99%	10.99%

**Table A6:** Safety level evaluation table.

	Low Safety	Moderate Safety	High Safety	Extremely High Safety
Grade score	50	67.5	82.5	95
Grade score range	40–60	60–75	75–90	90–100
